# A Peculiar Case of Resorption

**Published:** 1902-09

**Authors:** Louis Jack

**Affiliations:** Philadelphia


### A PECULIAR CASE OF RESORPTION.[1]

BY LOUIS JACK, D.D.S., PHILADELPHIA.

In the mouth of a woman of middle age the gingiva of a central incisor on the lingual aspect, and extending between this tooth and the lateral incisor, appeared inflamed, but without diffusion. This tissue was not patulous, as when salivary calculus exists, but was firm and slightly swollen, so that it extended somewhat lower on the lingual surface of the enamel than normal. This condition of the margin of the gum to a less extent had existed for at least a year. At the first observation it was supposed to have been caused by imperfect cleansing of the part. After instruction of the patient, as there was but little improvement of the condition, it was referred to impairment of nutrition associated with a marked gouty diathesis. On other occasions I had observed with gouty patients this peculiar state of the gingiva.

Subsequent exploration beneath the altered gingiva revealed a cavity supposed to be carious. After forcing the gum away by the expansion of a cotton pledget the cavity became sufficiently apparent to secure a careful examination, which proved it to be surprisingly deep, with a surface hard throughout and of extreme sensitivity. That this cavity had been produced by resorption of the cementum and dentine could be the only conclusion. The situation is nearly on the central portion of the lingual aspect extending in the apical direction four millimetres, transversely three and a half millimetres, with a depth of two millimetres. A considerable bay ex-

[1] Read before the Academy of Stomatology, Philadelphia, February 25, 1902.

tended under the enamel of the basilar ridge. This projecting enamel was broken down to improve access.

*Treatment.*—A speculum of copper was formed to correspond with the opening in the gum, which extended laterally somewhat beyond the cavity. This was so shaped that a ligature would bring it into apposition with the root at the sides and apical end of the resorbed area.

After arresting the bleeding with adrenalin, followed by alcohol and warmed air, the cavity was filled with archite, which was trimmed to conform with the root.

When it is considered that the root of this central is about five millimetres in the antero-posterior direction, the depth of this resorption endangered the pulp. It was nearly, if not entirely, exposed, as will directly be shown by the thermal reaction of that organ. On the first day of treatment the temperature-rate of the tooth was —66° +116° F. At the same time the normal rate proved to be —58° +130°, a range of tolerance of 72°. The tooth was also slightly intolerant of percussion and impatient of the passage of silk, these conditions being usually associated with thermal irritation of the pulp by heat so much above the normal as is shown here. The tone of color was a little deeper and duller than that of the other central, which is not uncommon in pulp disturbance. The bottom of the cavity had been capped with a celluloid disk covered with a paste composed of zinc oxide and eugenol.

The case at first was placed under observation each forty-eight hours, when application to the gum surface on the outer aspect above the tooth of tr. aconitum rad. and chloroform, āā, was made for twelve seconds at each interview.

TEMPERATURE TABLE OF CASES.

| | Normal rate. | Range of tolerance. |
|---|---|---|
| January 30 | —58° +130° F. | 72° |
| January 30 | —66° +116° F. | 50° |
| February 5 | —68° +118° F. | 50° |
| February 7 | +120° F. | |
| February 10 | —70° +123° F. | 53° |
| February 14 | --64° +124° F. | 60° |
| Tone of color improved. | | |
| February 18 | —64° +126° F. | 62° |
| February 21 | —63° +128° F. | 65° |
| February 24 | —60° +130° F. | 70° |
| Impatience to percussion much less. | | |

It will be observed that the range of tolerance steadily increased until it nearly reached that of the sound teeth.

The inflamed portion of the gingiva had been somewhat relieved by krameria diluted with water containing one grain to each ounce of zinc chloride.

In about a month after the discharge of the case the archite filling was found to be loose, when it was replaced with amalgam without further reaction of the pulp.

NOTE.—For the diagnostic value of pulp-reaction to thermal shock see *Dental Cosmos,* vol. xli. p. 1; "American Text-Book of Operative Dentistry," p. 406 *et seq.;* "Proceedings of Pennsylvania State Dental Society," for 1898.

